# Measurement of Iris Thickness at Different Regions in Healthy Chinese Adults

**DOI:** 10.1155/2021/2653564

**Published:** 2021-05-11

**Authors:** Qingchen Li, Yuan Zong, Huiming Wen, Jian Yu, Changbo Zhou, Chunhui Jiang, Guangxing Liu, Xinghuai Sun

**Affiliations:** ^1^Department of Ophthalmology and Vision Science, Eye and ENT Hospital, Fudan University, Shanghai 200031, China; ^2^Key Laboratory of Myopia of State Health Ministry and Key Laboratory of Visual Impairment and Restoration of Shanghai, Shanghai 200031, China; ^3^Zhejiang Putuo Hospital, Zhoushan 316100, Zhejiang, China; ^4^Suzhou Institute of Biomedical Engineering and Technology, Chinese Academy of Sciences, Suzhou 215163, China

## Abstract

**Purpose:**

To study the variation of iris thicknesses in different regions and explore the possible correlations with age and gender.

**Methods:**

Healthy Chinese adults were recruited; the anterior segment of their eyes was imaged by swept-source optical coherence tomography (SS-OCT). The horizontal scan of the right eye was selected, and the thicknesses of both the nasal and temporal irises were measured at 199 evenly spaced points.

**Results:**

A total of 233 subjects with an average age of 36.79 ± 10.04 years (range 19 to 62) were included in the final analysis. The mean iris thicknesses of the temporal and nasal sides were 364.79 ± 47.58 *μ*m and 372.44 ± 43.75 *μ*m, respectively. The mean nasal iris thickness was positively correlated with age (*β* = 0.9 *μ*m/year; *P* = 0.002), but the temporal one was not (*β* = 0.077 *μ*m/year; *P* = 0.806). At 139 points of the nasal iris and 146 points of the temporal iris, the iris thickness was significantly correlated with age (*P* < 0.05). The thicknesses of the peripheral and pupillary parts were positively correlated with age, while the middle part was negatively correlated with age. No significant difference was observed in the mean iris thickness between genders (temporal: *t* = 1.597, *P* = 0.112; nasal: *t* = 1.870, *P* = 0.063), but females had a thicker iris than males at 50 points in the temporal side and 49 points in the nasal side (*P* < 0.05); no point in males was observed to have thicker iris compared to females.

**Conclusion:**

Using SS-OCT and the novel method, thicknesses of the iris at different regions were measured. The thicknesses of the peripheral and pupillary irises increase with age, while the thicknesses of the middle part decrease.

## 1. Introduction

Aging is a natural process affecting almost all parts of the human body, including the eye. Studies had reported that the morphology of many important structures of the eye changes with aging. For example, it was reported that the mean corneal densitometry [1] and the axial and equatorial size of the crystalline lens [2] increase with aging, while the outer retinal thickness decreases [3].

The relationship between iris thickness and age remains controversial. Sun et al. found that the iris thicknesses measured at 500 *μ*m (IT500) and 750 *μ*m (IT750) from the scleral spur were positively correlated with age [4], while He et al. found a negative correlation between age and IT2000 [5]. One could easily see that different parts of the iris have been studied; this controversy could imply that the iris thickness at different points might change differently. As a result, it is interesting to have a more complete understanding of how the iris thickness changes with aging. Thus, we developed a new method that was able to measure the thickness of the object at 199 evenly spaced points; formerly, using this method, we studied the anterior chamber depth (ACD) at different regions of the anterior chamber. In this study, the iris thicknesses from 199 equally spaced points were measured and their potential correlation with age and gender was further explored.

## 2. Subjects and Methods

### 2.1. Subjects

Healthy Chinese volunteers were recruited from May to July 2015. All subjects were over 18 years of age and underwent a thorough examination including slit-lamp biomicroscopy, an undilated fundus examination by direct ophthalmoscopy, best-corrected visual acuity (BCVA), refraction measured by an autorefraction system, and the spherical equivalent (SE, diopter) calculated using the spherical diopter plus one-half of the cylindrical dioptric power, axial length (AL) measured by an IOL Master 500 (version 3.01; Carl Zeiss Meditec, Jena, Germany), and intraocular pressure (IOP) measured by a noncontact tonometer (Topcon CT-80A Computerized Tonometer; Topcon, Tokyo, Japan). The inclusion criteria were BCVA ≥16/20, IOP between 10 and 21 mmHg, SE between −3D and +1 *D*, and AL between 21 and 25 mm. Additionally, patients were excluded if they were using ocular topical medications at the time of enrollment or during the previous three months and had systemic conditions/treatments known to affect the iris, for example, application of a_1A_-receptors blockers that could lead to irreversible iris smooth muscle atrophy [6]. The exclusion criteria were BCVA <16/20; AL >25 mm or <21 mm; IOP >21 mmHg or <10 mmHg; history of ocular surgery; trauma or laser treatment; history of any intraocular disease; and family history of glaucoma in a first-degree relative. The present study was approved by the Institutional Review Board of the Eye and ENT Hospital, Fudan University, and was performed in accordance with the faith of the Declaration of Helsinki. All subjects provided informed consent.

### 2.2. Swept-Source OCT Imaging and Analysis

OCT scans were obtained using a commercially available SS-OCT system (CASIA SS-1000; Tomey Corporation, Nagoya, Japan; software version 6H.4) by a single observer under consistent light conditions (≈340 lux). Subjects were directed to remain in the primary gaze position towards an internal fixation light. The eyelids were kept open by a second examiner, who took caution to avoid placing any pressure on the eye. The standard anterior segment protocol, composed of 128 radial scans (each 16 mm in length and 6 mm in depth) within approximately 2.4 seconds, was used. Both eyes were imaged; during further analysis, only horizontal (0°–180°) scans from the right eyes were selected.

### 2.3. Measurement of Iris Thickness

**Figure 1:**
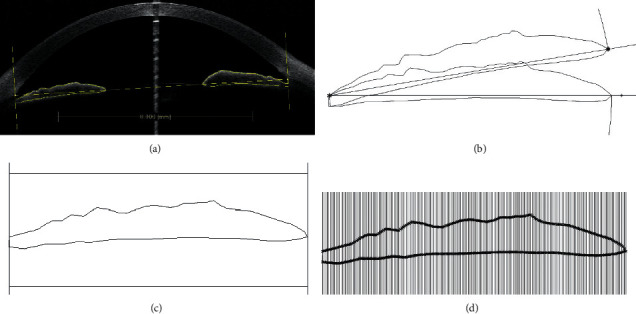
Measurements of iris thickness at 199 points. (a) Scleral spurs (SS) at both sides were identified; vertical lines through the SS that were perpendicular to the lines connecting the two SS, together with the anterior and posterior iris surface, defined the border of the iris. (b) After the background bitmap was removed, the iris was rotated so that the line connecting the end of the lateral anterior iris surface and pupillary margin (^∗^) became horizontal. (c) The image was recalibrated, and two horizontal lines parallel to the line connecting the anterior iris root and pupillary margin were drawn above and below the iris as references. (d) The iris was then divided into 200 sections by 199 equally spaced vertical lines across the iris with a specially edited path in the Grasshopper plug-in. The iris thickness was defined as the length of the vertical line between the anterior and posterior borders of the iris, point 1 referred to the first location from the lateral side, point 2 was the second, and the rest was deduced by analogy.

Rhinoceros NURBS Modeling for Windows (version 5.0; McNeel North America, Seattle, WA, USA) and the Grasshopper plug-in for the Rhino program (McNeel North America, Seattle, WA, USA) were used to measure the iris thickness (1–199). First, a scale bar was set on the OCT image using the built-in software, and the image was then exported. Second, the scleral spur (SS) on both sides were identified using the standard by Sakata, that is, a change in curvature on the inner surface of the cornea and an inward protrusion of the sclera [7]. Then, a line perpendicular to the line connecting the two SS was drawn through the SS at each side; this, together with the anterior and posterior surfaces of the iris, defined the boundary of the iris (Figure 1(a)). After that, the background bitmap was removed, and the outline of the iris was rotated so that the line connecting the lateral end of the anterior iris surface and the pupillary margin was on one horizontal line (Figure 1(b)). The image was then recalibrated, and two horizontal lines parallel to the line connecting the iris root and pupil margin were drawn above and below the outline of the iris (Figure 1(c)). The iris was then divided into 200 sections by 199 equally spaced vertical lines across the iris with a specially edited path in the Grasshopper plug-in (Figure 1(d)). The iris thickness was defined as the length of the vertical line between the anterior and posterior borders of the iris, point 1 referred to the first location from the lateral side, point 2 was the second, and the rest was deduced by analogy.

### 2.4. Repeatability and Reproducibility

The first 20 eyes were selected to test repeatability and reproducibility. Intraobserver repeatability was determined by one observer who measured points 1–199 twice, and interobserver reproducibility was evaluated by two observers who measured points 1–199 independently. Values from 0.81 to 1.00 for ICC indicate almost perfect agreement, while values less than 0.40 indicate poor to fair agreement.

### 2.5. Statistical Analysis

All analyses were performed using SPSS software version 26.0 (SPSS, Inc., Chicago, USA). Data are presented as the mean ± standard deviation (SD). The difference in iris thicknesses between genders was assessed by an independent-samples *T* test. A linear regression model was used to analyze the association between age (independent variables) and iris thicknesses at 199 points (dependent variable), and the unstandardized regression coefficients (*β* value) were recorded to assess the mutuality intensity. The level of significance was set at a *P* value of less than 0.05.

## 3. Results

Among the 309 subjects recruited, 233 (75.4%) were included in the final analysis. Seventy-six were excluded because one SS (67, 21.68%) or the bilateral (9, 2.91%) SS could not be identified. Among these 233 subjects, 114 were male (48.9%) and 119 were female (51.1%). The average age was 36.79 ± 10.04 years (evenly distributed from 19 to 62, Supplementary Figure S1); males and females had a similar age distribution (*t* = 0.215; *P* = 0.830).

The intraobserver and interobserver ICCs for the iris thickness measurements were greater than 0.80 for all 199 points on both sides. The intraobserver ICCs were greater than 0.9 at 151 points, and the interobserver ICCs were greater than 0.9 at 120 points for the temporal iris; for the nasal side, the intraobserver ICCs were greater than 0.9 at 156 points and interobserver ICCs at 126 points, respectively.

**Figure 2:**
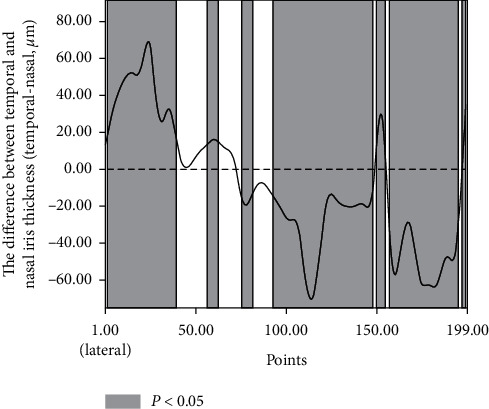
Differences between temporal and nasal iris thicknesses. Filled: the difference was statistically significant (*P* < 0.05).

The mean iris thicknesses of the 199 points in the temporal and nasal sides were 364.79 ± 47.58 *μ*m and 372.44 ± 43.75 *μ*m, respectively. The nasal and temporal irises had similar mean thicknesses, but the nasal iris was thicker than the temporal iris at 101 points and thinner at 54 (all *P* < 0.05) (Figure 2).

**Figure 3:**
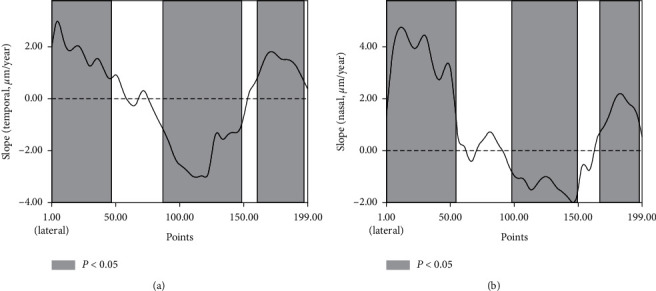
Variation of iris thickness with age (*μ*m/year): temporal iris (a) and nasal iris (b). Filled: the correlation was statistically significant (*P* < 0.05).

Univariate linear regression analysis found that the mean nasal iris thickness was positively correlated with age (*β* = 0.9 *μ*m/year; *P* = 0.002), but the temporal iris one was not (*P*=0.806). At 139 points of the nasal iris and 146 of the temporal iris, the iris thickness was significantly correlated with age (*P* < 0.05), and iris thicknesses from the peripheral (nasal: 1–55; temporal: 1–46 and 51) and pupillary (nasal: 166–197; temporal: 160–196) parts were positively correlated with age, while the middle part (nasal: 98–149; temporal: 87–148) was negatively correlated with age (Figure 3 and Supplement Figure S2).

**Figure 4:**
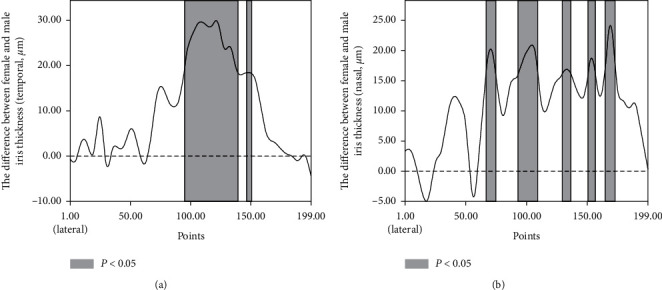
Iris thickness differences between females and males at the temporal (a) and nasal sides (b). Filled: the difference was statistically significant (*P* < 0.05).

Males and females had similar mean temporal (male 359.72 ± 46.06 *μ*m; female 369.65 ± 48.68 *μ*m; *t* = 1.597, *P* = 0.112) and nasal (male 366.99 ± 39.06 *μ*m; 377.66 ± 47.40 *μ*m; *t* = 1.870, *P* = 0.063) iris thicknesses, but females had a thicker iris than males at 50 points in the temporal iris and 49 in the nasal iris (*P* < 0.05); these points were primarily located at the middle part of the iris, and at no points were females observed to have a thinner iris than males (Figure 4).

## 4. Discussion

Using a novel method with good repeatability and reliability, the iris thickness at 199 evenly spaced points was successfully measured, and the possible correlation of iris thickness with age and gender was analyzed. The thicknesses of the peripheral and pupillary irises increased with age, while the middle part decreased. As a noncontact method performed in the sitting position, anterior segment optical coherence tomography (AS-OCT) is patient-friendly and used widely to study the iris thickness. Recently, with the introduction of swept-source OCT, which has improved scan speeds and axial resolution [8], an increasing number of studies have focused on the thickness and volume of the iris [9–12]. Invernizzi et al. found that iris color was associated with iris thickness [13], and others also reported a correlation between furrows, crypts [10], and ethnicity [14] and iris thickness. Previous studies have reported that iris volume (IV) was not affected by aging [13, 15]. Theoretically, the unchanging IV and increasing SSL-to-PM or AISL would result in a decreasing iris thickness. However, studies on iris thickness have controversial results. Sun et al. found that IT500 and IT750 were positively correlated with age [4], while He et al. found that IT2000 was negatively correlated with age [5]. These results seemed to suggest that the iris thickness at different locations might change differently with age. Our former study has established a method to evaluate the depths of the anterior chamber at 199 different points, and the annual reduction percentage of ACD with age was found to be higher at peripheral and central regions but lower at middle peripheral region, which corresponded with the root, the pupillary margin, and the middle part of the iris, respectively [16]. In the present study, the thicknesses from 199 evenly located points of the iris were studied by the same method. At 139 (87 positive and 52 negative) points of the nasal and 146 (84 positive and 62 negative) of the temporal sides, the iris thickness was significantly correlated with age (*P* < 0.05); the thicknesses of the peripheral and pupillary irises increased with age, while that of the middle part decreased, and the thickness of the transiting area between them did not change significantly with age.

The reason for different changes of iris thickness at different parts is unclear, but the following might be the cause. Histopathological studies showed that the major arterial circle was located at the iris root and the minor vascular circle was located at the iris margin; the middle iris is mostly composed of loose connective tissue containing fibroblasts, which is the primary cell type that produces collagen [17]. With age, the fibroblasts and collagen fiber in connective tissue were found to decrease throughout the body [18, 19], while the thickness of the arterial adventitia increased [20]. On the other hand, “Koganei” cells, which are round and oval clumps with a nodular, mulberry-like appearance that are filled with clusters of melanin granules, were primarily found near the pupillary border and iris root. Koganei cells, as well as the melanin granules inside of them, increased in number with age [21]. Wobmann speculated that this is because there is a slight but continuous breakdown of pigmented cells throughout life. The pigment granules released from the iris epithelium first contact the iris stroma in the pupillary region and then are carried toward the drainage angle, where they are taken up by Koganei cells. The difference in the organizing tissue and accumulation of Koganei cells might contribute to the difference in iris thickness changes with age.

The increasing iris thickness in the peripheral and pupillary parts and decreasing iris thickness in the middle part with age might lead to the increasing incidence of primary angle-closure glaucoma (PACG) despite the insignificant change in iris volume with age. The current mainstream mechanisms of PACG were divided into four types using AS-OCT: pupillary block (PB), plateau iris configuration (PIC), thick peripheral iris roll (thick PIR), and exaggerated lens vault [22, 23]. The increasing iris thickness at the pupillary and iris root parts with aging might contribute to the pathogenesis of PD or thick PIR, respectively. Our findings are consistent with the high prevalence of PACG in elderly populations.

It was also noticed that the average thickness of the nasal iris increased with age, while that of the temporal iris did not. In both the temporal and nasal sides, the thickness at the periphery and pupillary parts was positively correlated with age, while the middle was negatively correlated. However, we found that the mean reduction rate of the middle part (points 56–159) was steeper at the temporal side than at the nasal side, while the slope of increasing thickness was higher in the nasal peripheral (points 1–55) than in the temporal peripheral (*P* < 0.05, Supplementary Table S1 and Figure S3) with age. The slower rate of reduction at the middle and higher increase at the periphery of the nasal iris might explain that while the mean nasal iris thickness was positively correlated with age, the temporal side was not. Furthermore, the thickening of the nasal iris with age was in accordance with the clinical impression that the anterior chamber angle was narrower in the nasal than in the temporal sides. Previously, Tun et al. reported that the anterior chamber angle was narrower in the nasal than in the temporal sides in elderly subjects [24]. In former reports by Cheon et al. [25] and Maruyama et al. [26], one could also notice that, with aging, the nasal anterior chamber angle area reduced more quickly than that of the temporal side. Our finding that the nasal iris, especially the peripheral part, thickened primarily with age supports these reports and partly explains the cause.

In the present study, only the horizontal scan was studied; studies have previously reported that the superior and inferior anterior chamber angles might be slightly different from those in temporal and nasal quarters [27, 28]. Future studies should take this into consideration. Moreover, all the subjects enrolled in the present study were healthy Chinese; studies including healthy subjects with other ethnic backgrounds and PACG eyes might be able to reveal additional findings.

The patterns found here in physiological condition are essential for pathophysiological study, and the observation of this study should be the foundation for subsequent research studies. Secondly, the present result showed that age has a differential effect on the different segments of the iris, and in future exploring of the potential role of age in the iris-related PACG, the location of the iris should be of importance. Also, the novel way of image processing could be applied to the study of others.

## 5. Conclusions

In conclusion, iris thicknesses from different parts of the iris were successfully measured by the novel method described here. Iris thicknesses at different points changed differently with age. The thicknesses at the pupillary margin and root increased, while that of the middle part decreased.

## Data Availability

The data used to support the findings of this study are available from the corresponding author upon request.

## Supplementary Materials

Supplementary Materials Supplementary Table S1: difference in the changing of iris thickness with age between temporal and nasal. Supplementary Figure S1: the age distribution of subjects. A total of 233 subjects with an average age of 36.79 ± 10.04 years were enrolled, and the age evenly distributed from 19 to 62. Supplementary Figure S2: the direct linear correlation between age and iris thickness. The scatter plots demonstrated the correlation between age and thickness of the peripheral, middle, and pupillary parts of the temporal and nasal sides. Supplementary Figure S3: changes in the nasal/temporal iris thickness with age at three regions. The reduction rate of the middle part (points 56–159) was steeper at the temporal side (the dotted line) than at the nasal side (the solid line), while the slope of increasing thickness with age was higher at the nasal peripheral (points 1–55) than at the temporal peripheral.
