# Supplementary material for: Measurement of Iris Thickness at Different Regions in Healthy Chinese Adults
Source: J Ophthalmol. 2021 May 11;2021:2653564. doi: 10.1155/2021/2653564 (PMC8131156; doi:10.1155/2021/2653564)
Supplement: Supplementary Materials — Supplementary Table S1: difference in the changing of iris thickness with age between temporal and nasal. Supplementary Figure S1: the age distribution of subjects. A total of 233 subjects with an average age of 36.79 ± 10.04 years were enrolled, and the age evenly distributed from 19 to 62. Supplementary Figure S2: the direct linear correlation between age and iris thickness. The scatter plots demonstrated the correlation between age and thickness of the peripheral, middle, and pupillary parts of the temporal and nasal sides. Supplementary Figure S3: changes in the nasal/temporal iris thickness with age at three regions. The reduction rate of the middle part (points 56–159) was steeper at the temporal side (the dotted line) than at the nasal side (the solid line), while the slope of increasing thickness with age was higher at the nasal peripheral (points 1–55) than at the temporal peripheral. [file 2653564.f1.zip › 2653564.f1/Supplementary Figure S3.pdf]

Supplementary Figure S3. Changes in the nasal/temporal iris thickness with age at three regions.

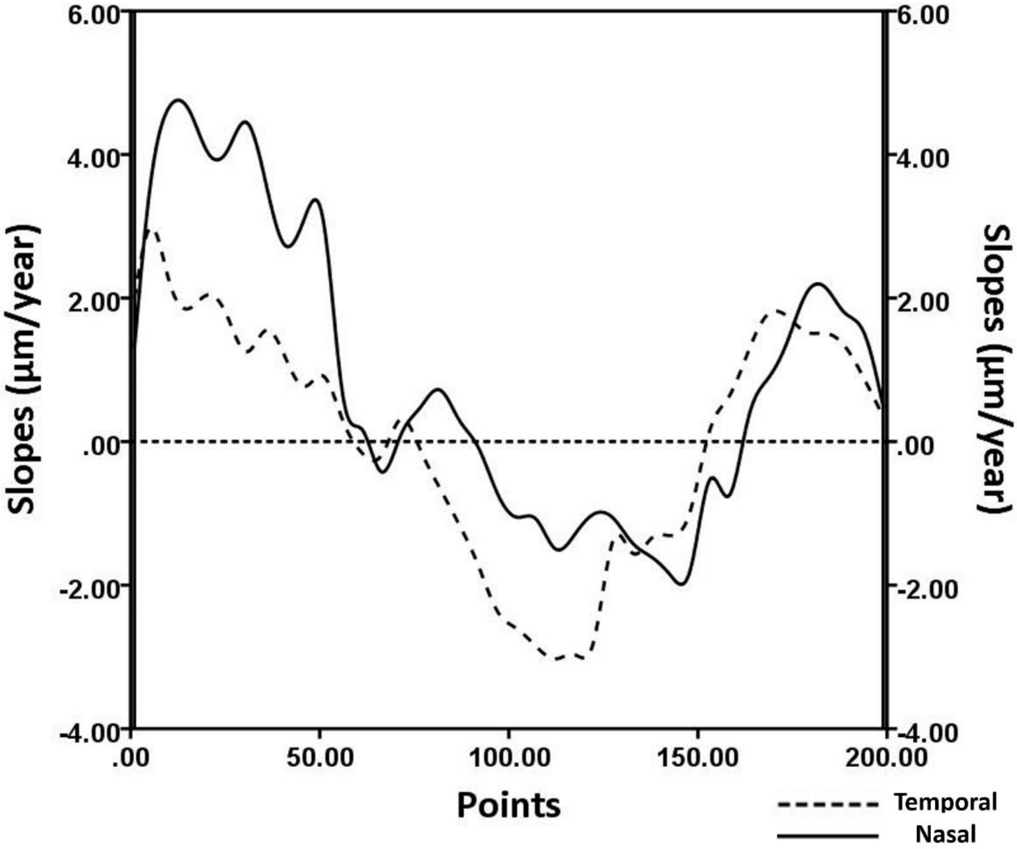

The reduction rate of the middle part (points 56-159) was steeper at the temporal (the dotted line) than nasal sides (the solid line), while the slope of increasing thickness with age was higher at the nasal peripheral (points 1-55) than at the temporal peripheral.
